# Editorial: Tuberculosis and humoral immunity

**DOI:** 10.3389/fimmu.2025.1562567

**Published:** 2025-02-10

**Authors:** Taru S. Dutt, José Alberto Choreño-Parra

**Affiliations:** ^1^ Department of Microbiology, Immunology, and Pathology, College of Veterinary Medicine and Biomedical Sciences, Colorado State University, Fort Collins, CO, United States; ^2^ Departamento de Enseñanza, Instituto Nacional de Enfermedades Respiratorias Ismael Cosío Villegas, Mexico City, Mexico

**Keywords:** tuberculosis, mycobacterium, B cells, antibodies, protection, diagnostics

Tuberculosis (TB), caused by *Mycobacterium tuberculosis* (Mtb), remains a formidable global health threat, afflicting nearly one-quarter of the world’s population. A complete understanding of the immune components involved in natural protective defenses against Mtb is a crucial step to control this epidemic. Unfortunately, we are still lacking effective therapies and vaccines that confer sterilizing immunity, despite decades of immunology and TB research co-development. The parallel progress of the two sciences allowed historical advances in one area to have great impact on the other and vice versa. For instance, much of what constituted the foundation of cellular immunology was based on Mtb as the ideal model for studying delayed-type hypersensitivity.

As a result, observations derived from the TB field are typically generalized to other areas of immunopathology and vaccinology. One of such generalization is the assumption that T cell responses (a very destructive weapon of the immune system) are the chief protective mechanism against intracellular bacteria. Thus, TB research has emphasized the role of cell-mediated immunity (CMI), particularly CD4+ and CD8+ T cells, in combating Mtb (1–14). However, vaccine candidates designed to promote class one T helper (Th1) cell responses have failed to control this infection (15–17), suggesting that other components of the immune system are essential for protection. Indeed, emerging evidence (18–27), including our own findings (28–30), is reshaping this perspective by underscoring the critical contributions of humoral immunity in host defense against TB.

This editorial synthesizes the current understanding of humoral immunity in TB, its potential to enhance diagnostics and vaccine strategies, and the interplay between cellular and humoral responses. Our aim was to provide a collection of selected manuscripts contributing to clarify the misconception that B cells and antibodies are ineffective against intracellular pathogens like Mtb, an assumption that has been challenged by recent studies demonstrating that humoral immunity is not only relevant but also potentially protective in TB.

A provoking reflection necessary to challenge current dogmas in TB research is the idea of tissues as crucial orchestrators of the class of immune response deployed against invading microorganism (31). As such, the lungs must determine the ultimate combination of effector mechanisms more suitable to ensure bacilli clearance while preserving the local architecture of the tissue. With this in mind, what is the benefit of evoking such a destructive stereotyped response like CMI within a local microenvironment so delicate to allow the vital function of gas exchange? Perhaps other immune components at the site of encounter with Mtb [including mucosal antibodies and B cells of bronchial-associated lymphoid tissue (BALT)] are better at controlling the infection while preserving the structure of the lungs due to their less harmful effects.

B cells, for instance, can act as antigen-presenting cells, modulate T cell responses, and produce antibodies that neutralize Mtb or mediate effector functions. Notably, B-cell aggregates in Mtb-infected lungs have been correlated with reduced bacterial burden and improved outcomes of TB disease (20, 23, 30). Antibodies have been shown to enhance macrophage uptake and intracellular killing of Mtb via Fc receptor-mediated pathways (18, 32). Furthermore, Mtb-specific IgA and IgG antibodies contribute to mucosal immunity and bacterial clearance in experimental models (33, 34).

The research highlighted in this Research Topic further emphasizes some of these ideas and provide proof-of-concept evidence. First, Flores-Gonzalez et al. demonstrated distinct alterations in B cell subsets and reduced cytokine production, such as IFN-γ and IL-10, in patients with active TB, particularly drug-resistant cases. These findings suggest that phenotypical alterations of B cells may serve as readouts of the failure of lung tissue-instructed defenses to eliminate Mtb. Also, in their review article, McIntyre et al. highlighted increasing evidence supporting the role of antibody-mediated immunity in TB, demonstrating that antibodies contribute to pathogen neutralization, promote phagocytosis, and mediate antibody-dependent cellular cytotoxicity (ADCC). These effector mechanisms of antibodies might be pivotal at the early stages of the disease when first contact with Mtb occurs. Especially during early childhood and adolescence, social factors like breastfeeding, undernutrition, hygiene, poverty, exposure to environment mycobacteria, and respiratory infection history might influence the repertoire of antibodies available in the respiratory tract. Thus, analyzing humoral immunity to TB in distinct age groups is of significant relevance, as here represented by studies on maternal and infant immunity by Hjelmar et al., which suggest that antibodies against Mtb antigens, such as lipoarabinomannan (LAM), may offer protection against disease progression during early childhood. Although significant progress has been made, the diversity of TB-specific antibodies presents challenges for vaccine and diagnostic development. Advances in deep-learning models can improve the high-throughput analysis of B cell receptors, revealing maturation pathways and functional diversity, which could improve vaccine design and diagnostics (Lee et al.).

Despite these promising findings, several questions remain. First, what are the mechanisms underlying antibody-mediated protection against Mtb. Second, what are the antibody-independent roles of B cells in TB? Finally, how can we unravel the intricate crosstalk between humoral and cellular immunity to inform the design of more effective vaccines and therapies?

In conclusion, the role of humoral immunity in TB represents a paradigm shift that challenges the traditional focus on CMI. While Th1 cells remain central to controlling Mtb infection, antibodies, and B cells play complementary roles that cannot be ignored. The integration of humoral responses into TB vaccine design, diagnostics, and therapeutics holds immense potential for improving disease management and outcomes. Future research should prioritize characterizing antibody and B cell repertoires in lung specimens of TB patients, uncovering the mechanisms by which antibodies mediate protection, identifying key antigenic targets, and leveraging these insights to develop novel interventions.

As we continue to unravel the complexities of the immune response to TB, a more comprehensive approach that incorporates both cellular and humoral immunity will be essential. Such an approach not only broadens our understanding of TB pathogenesis but also paves the way for more effective vaccines and therapies, ultimately bringing us closer to the goal of TB eradication.
